# Foliar Spray with Vermiwash Modifies the Arbuscular Mycorrhizal Dependency and Nutrient Stoichiometry of Bhut Jolokia (*Capsicum assamicum*)

**DOI:** 10.1371/journal.pone.0092318

**Published:** 2014-03-20

**Authors:** Mohammad Haneef Khan, Mukesh K. Meghvansi, Rajeev Gupta, Vijay Veer, Lokendra Singh, Mohan C. Kalita

**Affiliations:** 1 Defence Research Laboratory, Defence Research & Development Organisation, Post Bag 2, Tezpur, Assam, India; 2 Directorate General of Life Sciences, Defence Research & Development Organisation, New Delhi, India; 3 Department of Biotechnology and Bioengineering, Gauhati University, Guwahati, Assam, India; Centro de Investigación y de Estudios Avanzados, Mexico

## Abstract

Vermiwash (VW), a liquid extract obtained from vermicomposting beds, is used as an organic fertilizer for crop plants. The current study investigated the effect of a vermiwash foliar spray on the response of bhut jolokia (*Capsicum assamicum*) exposed to two different arbuscular mycorrhizal fungi (AMF*: Rhizophagus irregularis,* RI and *G. mosseae,* GM) in acidic soil under naturally ventilated greenhouse conditions. The VW spray significantly influenced the growth of plants receiving the dual treatment of AMF+VW. Plant growth was more prominent in the GM+VW treatment group than that in the RI+VW treatment group. The plant-AMF interactions in relation to growth and nutrient requirements were also significantly influenced by the application of VW. Interestingly, the VW treatment appeared to contribute more N to plants when compared to that under the AMF treatment, which led to changes in the C:N:P stoichiometry in plant shoots. Furthermore, the increased potassium dependency, as observed in the case of the dual treatments, suggests the significance of such treatments for improving crop conditions under salt stress. Overall, our study shows that the VW foliar spray modifies the response of a crop to inoculations of different AMF with regard to growth and nutrient utilization, which has implications for the selection of an efficient combination of nutrient source for improving crop growth.

## Introduction

Vermiwash (VW) is a brownish-red liquid extract collected during vermicomposting of organic waste. VW can also serve as a valuable foliar spray, because it is composed of excretory products and mucus secretions from earthworms and micronutrients from the organic molecules in the soil. These nutrients are absorbed and then transported to the leaves, shoots, and other parts of a plant [1]. Various experimental results have shown that the application of VW or compost tea improves plant health, yield, and nutritional quality [2], [3]. Studies have shown that vermicompost tea contains plant growth-promoting substances (e.g., humic, fulvic, and other organic acids [4]; auxin-like substances [5]; and cytokinin-like substances [6]). A VW foliar spray is more advantageous from economical and environmental perspectives owing to the absence of nutrient leaching, which is often encountered when performing soil amendments.

Arbuscular mycorrhizal fungi (AMF), the most widespread root fungal symbionts associated with the vast majority of higher plants, have been shown to increase plant growth and yield by improving nutrient uptake in various agroecosystems [7], [8]. In addition, AMF have been reported to enhance stress tolerance in plants under severe stress conditions, including heavy metal, drought, and salinity [9]. Although AMF species, typically, are considered nonspecific, variations in the growth response of different plant genotypes to different AMF species have been described [7], [8]. Such variation in the growth response, termed mycorrhizal species sensitivity, implies that the mycorrhizal dependency of plants can also vary [10]. The determination of plant growth responses under controlled conditions, therefore, is a valuable first step in evaluating the importance of symbiotic relationships in nature and understanding the dynamic interactions within an ecosystem [11].

In intensive agriculture systems, major emphasis has been placed on increasing the input of nutrients through various approaches to achieve higher crop productivity, thus, leading to low nutrient-use efficiency and increased environmental problems. The chemical and biological processes that occur in the rhizosphere affect the mobilization and acquisition of soil nutrients and microbial dynamics; they also control the nutrient-use efficiency of crops, and therefore, profoundly influence crop productivity and sustainability [12]. Thus, researchers indicate that it is necessary to maintain greater nutrient-use efficiency through the integration of various approaches, including the combined use of organic amendments and chemical fertilizers [13] or organic amendments and AMF [14], [15]. The majority of these studies have provided empirical evidences on the usefulness of such approaches with regard to soil amendments [13], [15]. However, to the best of our knowledge, no reports are available on the effects of the combined use of VW, as a foliar spray, and AMF, as soil inoculants. In addition, the influence of such amendment methods on each other could be exacerbated when applied to acidic soils that have high phosphorus (P)-fixing power because of the excessive presence of iron and aluminum ions, thus, resulting in low P availability for crop production [16].

Carbon (C), Nitrogen (N), and P are the major plant nutrients that are both structurally and functionally essential to all organisms [17]. A mechanistic link between tissue elemental stoichiometry (C:N:P *per se*) and the growth rate of an organism has been suggested [18], [19]. P is a critical element in the production of ribosomes; ribosomes manufacture N-rich proteins, which in turn, constitute the C and energy-harvesting organs [20]. The growth rate hypothesis proposes that higher growth rates are associated with lower C:N, C:P, and N:P ratios. Therefore, variations in the P levels in organisms are driven by differences in the allocation of P-rich ribosomal RNA (rRNA), which is required to meet the protein synthesis demands of increased growth rates [17], [18], [21]. Thus, the factors or physiological changes that affect growth rates could result in differences in the C:N:P ratio of biomass and cellular allocation [21]. The combined use of biological and organic amendments may differentially affect a crop with respect to nutrient-use efficiency and C:N:P stoichiometry, thus, affecting the potential benefits and limitations. For instance, the excessive use of organic sources of P can result in the suppression of the AMF community [22], thereby highlighting the significance of careful nutrient budgeting for optimizing, rather than maximizing, soil fertility.

Crop management involves a range of practices that can affect AMF associations directly (e.g., by damaging or killing AMF) and indirectly (e.g., by creating favorable or unfavorable conditions for AMF) [23]. The organic sources of nutrients (e.g., farmyard manure, compost, and crop residues) and slow release of mineral fertilizers (e.g., rock phosphate) do not typically suppress AMF and may even stimulate their growth [24]. However, the overuse of organic amendments, especially those high in P, such as chicken manure, may have a negative influence on AMF, and the precise effect of the organic amendments has been shown to be variable (i.e., dependent on the type of soil or amendment) [22]. Therefore, the role and extent of the effects of a combination of organic and biological amendments on crops should be investigated to obtain more precise data before such practices are implemented in crop fields. Furthermore, it is imperative to determine the extent to which organic amendments affect the response of a crop to different AMF species. The present study was undertaken with the objective of determining the influence of a VW foliar spray on mycorrhizal growth dependency and nutrient stoichiometry of bhut jolokia (*Capsicum assamicum*) inoculated using two different AMF, in acidic soil under pot conditions. Bhut jolokia is a highly pungent chili species native to the northeastern region of India and has tremendous ethnopharmacological potential [25].

## Materials and Methods

### Plants, AMF and vermiwash

Three-week-old seedlings of bhut jolokia procured from a local nursery (M/s Lakshmi Agric. Pabohi, District Sonitpur, Assam, India) were used in the pot experiment. The pure inocula of two AMF (i.e., *R. irregularis* and *G. mosseae*) were supplied by The Energy and Resources Institute (TERI), New Delhi. The AMF bulk inoculum preparation was conducted in a naturally ventilated greenhouse by sowing surface-sterilized maize (*Zea mays* L.) seeds in earthen pots with an autoclaved soil:sand mixture (1:1, w/w). The substrate containing the spores and root pieces served as the stock culture for AMF inoculum. The VW was prepared from air-dried mixed waste (i.e. vegetable waste:paddy straw:water hyacinth; 1:1:1;) according to the procedure suggested by Ismail [26] using a cylindrical metal container (30-cm height ×30-cm diameter). In brief, a tap was fixed on the lower side of the container. The bottom of the container had a 5-cm layer of broken pebbles, followed by a 5-cm layer of coarse sand. Air-dried mixed waste as mentioned above was put into the container as substrate and one thousand earthworms (*Eisenia fetida* Savigny) were introduced into the substrate. An appropriate level of moisture (60%) was maintained in the container by adding water at regular intervals. During the VW preparation, the temperature and pH inside the container ranged from 29–32°C and 7.0–7.3 respectively. The brown-colored watery extract of earthworm-worked substrate was allowed to drain out of the container and used for foliar spray on test crops. The physico-chemical composition of the VW is presented in Table 1.

**Table 1:** Physico-chemical properties of the soil and vermiwash used in the study.

	Soil		Vermiwash
pH (H_2_O)	6.330	±0.072*	8.77
Electrical conductivity (dS m^−1^)	0.027	±0.009	0.04
Available Nitrogen (mg kg^−1^)	350.816	±21.831	338.88**
Available Phosphorus (mg kg^−1^)	55.041	±0.120	57.2**
Available Potassium (mg kg^−1^)	314.27	±19.548	154.94**
Total Organic Carbon (%)	1.160	±0.027	1.06

*Mean ± standard error (n = 3). **Values in ppm.

### Pot experiment

The pot experiment was performed from 6 May to 5 August 2011, in a naturally ventilated greenhouse at the Defence Research Laboratory (DRL), Tezpur (26°38′N, 92°48′E), District Sonitpur, Assam. Air-dried and sieved soil collected from the experimental field of the DRL was used to fill earthen pots (3.5 kg pot^−1^). The physico-chemical composition of the experimental soil is presented in Table 1. Ten grams of mycorrhizal inoculum (containing 20 spores per g) of each AMF (i.e., *R. irregularis* and *G. mosseae*) was placed in each pot (2 cm below the seed sowing level) and covered using soil. Initially, five seedlings were grown in each pot, which were thinned to three at the start of the experiment. There were nine replicates of each treatment with a randomized block design. The plants in the pots were grown for 90 d in the greenhouse (temperature, 27–35°C; relative humidity, 70–80%) under natural illumination and watered (i.e., tap water) as needed.

### Physico-chemical analysis of the soil and VW used in the experiment

The pH of diluted-water (1:5) sample was determined using a digital pH meter (Model PT10; Sartorius AG, Germany). Electrical conductivity (EC) was determined using a conductivity bridge (Model 306; Systronics, India). Organic carbon (OC) was estimated using the method of Walkley and Black [27], by using 1N potassium dichromate and back-titrated using 0.5N ferrous ammonium sulfate solution. Available nitrogen (N) was determined by Kjeldahl method [28], by using an automatic nitrogen estimation apparatus (Model KelPlus Classic DX VA; Pelican Equipments, Chennai, India). Available P was determined spectrophotometrically in a 1M acidic ammonium fluoride solution [29]. Available potassium (K) was measured by the ammonium acetate method [30] using a flame photometer (Model FP114; Thermo Scientific, USA).

### Plant growth parameters

Plants were uprooted after 90 d, and the data on vegetative growth, chlorophyll content (SPAD), shoot nutrients, AMF colonization in the roots, and AMF spore count in rhizospheric soil were recorded. Shoot and root dry matters were recorded after drying the sample at 70°C for 48 h. The relative amount of chlorophyll present in the leaves was determined by measuring the transmittance of the leaf in two wave bands (i.e., 600–700 nm and 400–500 nm) by using a SPAD Meter (SPAD 502 Plus; Konica Minolta, Japan). SPAD values from the midpoint region near the midrib of five fully expanded leaf samples from a single plant were taken at 1100 IST, and the mean value was calculated [31].

### Shoot nutrients

For nutrient analysis of the shoots, the oven-dried samples were finely ground. Total N and C in the shoots were determined using an elemental analyzer (EA3000, Eurovector, Italy). To estimate the P, K, Na, and Ca levels in the shoots, 1 g of the finely ground sample was subjected to a wet oxidation treatment using tri-acid (HNO_3_:H_2_SO_4_:HClO_4;_ 10:1:4) digestion in a digestion block (KELPLUS, KES12L; Pelican Equipments, Chennai, India) at 200°C. Following acid digestion, the samples were diluted and filtered for further nutrient analysis. Total P in the shoots was determined by the vanadomolybdophosphoric acid colorimetric method [32] using a spectrophotometer (Specord 200; Analytik Jena, Germany). Total shoot Ca, Na, and K were measured by the ammonium acetate method of Hanway and Heidel [30], by using a flame photometer (Model FP114; Thermo Scientific, USA). For estimating mycorrhizal colonization, freshly collected roots were washed in water, cleaned using10% KOH, acidified using 1N HCl, and stained in 0.05% trypan blue [33].

### Mycorrhizal colonization and AMF spore count in rhizospheric soil

Quantification of root colonization for AMF was conducted using the gridline intersection method [34], and 100 segments from each sample were observed under a compound microscope (Leica DM750). Spores of AMF were extracted from the rhizospheric soil (50g for each treatment) before and after experimentation using the wet-sieving and decanting methods [35]. The total number of spores was estimated and the values were expressed as *x* number of AMF spores per 50 g of soil.

### Calculation of mycorrhizal dependency

Mycorrhizal dependency (MD) based on shoot dry matter was calculated according to the formula suggested by van der Heijden [36] as given below:
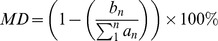



Where *a* is the mean total shoot dry matter in a given treatment (AMF and/or VW], *n* is the number of treatments, and *b* is the mean shoot dry matter of untreated plants. Similarly, the nutrient dependencies were calculated, where the values of a given shoot nutrient were used in place of the shoot dry matter in the above-mentioned formula.

### Statistical analysis of the data

Means were compared by ANOVA, followed by the least significant difference (LSD) test. Statistically significant differences were then determined at *p*  =  0.05, using the software IBM SPSS Statistics version 19.

## Results

### Mycorrhizal colonization and AMF spore count

**Figure 1:**
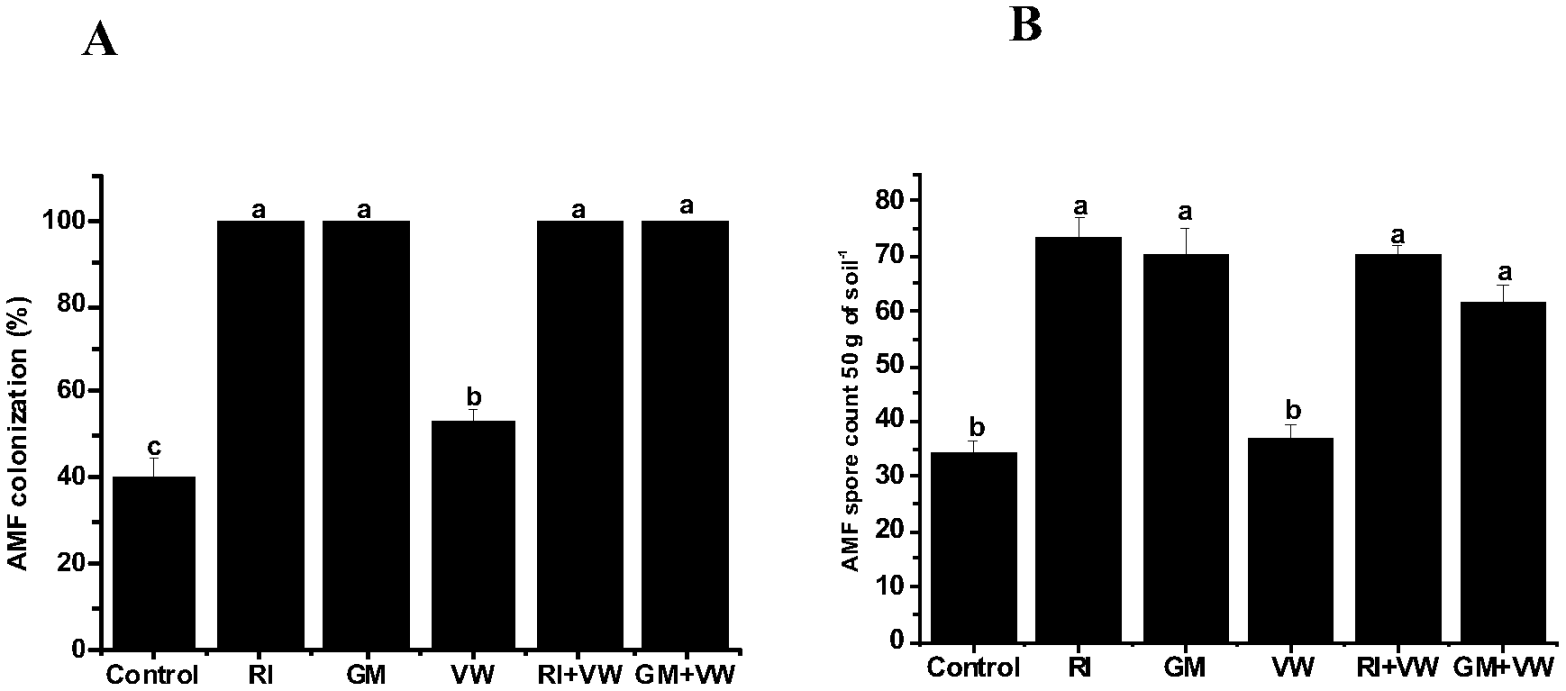
The AMF colonization in the roots (A) and spore count in rhizospheric soil (B). RI- *Rhizophagus irregularis,* GM- *G. mosseae,* VW- vermiwash. Values are means of nine biological replicates. Bar shows standard error of mean (SEM). Different letters on the column represent significant difference (*p*< 0.05) based on LSD test.

Root colonization (RC) at the beginning of the experiment was 26.66%, which increased up to 40% in the control plants following completion of the experiment. Furthermore, comparisons of the control to the AMF- and VW-treated plants suggests a statistically significant increase (*p*≤ 0.05) in RC; 100% RC was recorded in all the plants receiving the AMF treatments (Figure 1). Except for the single treatment with VW, there was no statistically significant difference with respect to RC among the single and dual treatments. Mycorrhizal spore count in the rhizospheric soil at the beginning of the experiment was 24.33 spores per 50 g^−1^, which increased to 34.33 in the control following completion of the experiment. Furthermore, a comparison of the control to the AMF- and VW-treated plants revealed a significant increase (*p*≤ 0.05) in spore count, except for the single treatment with VW. Comparisons among the AMF treatments did not show significant differences with respect to the spore count in the rhizospheric soil (Figure 1).

### Plant growth parameters and nutrient uptake

The shoot dry matter (SDM), root dry matter (RDM), and chlorophyll content increased significantly in the plants receiving single and combined treatments of AMF and VW in comparison to the control. However, the RDM:SDM ratio in all treated plants decreased significantly when compared to that in the control. Inoculations of RI increased the growth values (SDM and RDM) more than those of GM, and this increase was statistically significant. However, there were no significant differences between the RI and GM treatments with respect to the chlorophyll content and the RDM:SDM ratio. The pattern of growth observed for the three individual treatments followed the order RI>GM>VW. Overall, the growth values increased significantly in the dual treatments when compared to those in the single treatments. However, comparison of the RI and RI+VW treatments revealed a significant increase only in the RDM. In addition, the VW treatment considerably influenced the increase in growth of the plants exposed to dual treatments. The influence of VW on growth was more prominent in the GM+VW treatment when compared to that in the RI+VW treatment. With regard to the dual treatments, SDM, RDM, and chlorophyll content in the plants was higher under the GM+VW treatment when compared to those in the RI+VW treatment. However, a statistically significant difference in the growth parameters was noted only for the RDM and chlorophyll content (Table 2).

**Table 2:** Effect of foliar spray of vermiwash on response of inoculation of two different AM fungi in *Capsicum assamicum* under pot conditions.

Parameters	Control	*Rhizophagus irregularis*	*Glomus mosseae*	Vermiwash	*R. irregularis* +Vermiwash	*G. mosseae* +Vermiwash
Shoot dry matter (g)	1.390±0.074 d	3.529±0.062 ab	2.780±0.034 c	2.400±0.068 c	3.613±0.252 a	3.970±0.026 a
Root dry matter (g)	0.563±0.023 f	0.973±0.009 c	0.860±0.032 d	0.640±0.012 e	1.060±0.012 b	1.220±0.021 a
Root:shoot ratio	0.406±0.004 a	0.275±0.002 bc	0.308±0.013 b	0.268±0.006 c	0.296±0.017 b	0.307±0.003 b
Chlorophyll content*	30.400±0.740 e	35.90±0.309 bc	39.300±0.478 bc	35.233±0.165 bcd	38.233±0.320 b	44.933±2.045 a
Total shoot N (%)	2.856±0.023 abc	3.562±0.012 ab	3.432±0.023 abc	4.042±0.094 ab	4.673±0.198 a	4.959±0.549 a
Total shoot P (%)	0.139±0.002 d	0.226±0.008 ab	0.221±0.001 ab	0.177±0.007 abc	0.240±0.002 a	0.246±0.004 a
Total shoot K (%)	0.173±0.001 f	0.206±0.002 d	0.181±0.000 e	0.256±0.001 c	0.269±0.000 b	0.326±0.000 a
Total shoot C (%)	39.410±0.578 d	47.26±0.506 b	46.199±0.279 bc	44.938±0.175 bc	48.378±0.201 b	52.296±0.605 a
Total shoot Na (mg kg^−1^)	66.660±13.60 abc	150.00±23.571 a	135.00±6.236 ab	110.000±4.714 ab	161.667±3.600 a	193.333±5.443 a
Total shoot Ca (mg kg^−1^)	566.660±13.60 d	800.00±23.571 c	766.66±13.609 c	750.000±23.571 c	883.33±13.609 b	1116.667±13.609 a

Values are means ± standard errors of nine biological replicates. Values within a column not followed by the same letter are statistically.

different (p<0.05). *Measured as SPAD value using chlorophyll meter (SPAD 502 Plus; Konica Minolta, Japan).

N- Nitrogen; P- Phosphorus; K- Potassium; C- Carbon; Na- Sodium; Ca- Calcium.

In comparison to the control plants, plants under single and combined treatments of AMF and VW showed significantly higher nutrient (N, C, P, K, Na, and Ca in the shoots) uptake. However, the concentration of N in the shoots of plants exposed to the single treatments of RI and GM did not significantly differ from that in the control. A comparison between the RI- and GM-inoculated plants indicated that the concentration of K in the shoots was significantly higher in the RI-inoculated plants but, there were no significant differences in the concentrations of the other shoot nutrients. The maximum concentration of K in the shoots was observed in the plants receiving the VW treatment; this finding was statistically significant when compared to the plants receiving the single AMF treatments. However, the plants receiving the VW treatment had significantly lower P when compared to that in the GM- and GI-treated plants. The maximum total C in the shoots was observed in the RI-treated plants, followed by the GM- and VW -treated plants. However, among the single treatments, a significant difference with respect to total C was found only between the RI- and VW-treated plants. A comparison of the RI and RI+VW treatments revealed a significant increase only in the K, Ca, and N contents in the shoots. However, all of the shoot nutrients were significantly higher in the GM+VW-treated plants when compared to those in the GM-treated plants. In addition, while comparing the VW treatment to the GM+VW and RI+VW treatments, the uptake of all shoot nutrients was shown to increase significantly. However, the concentration of N in the shoots of the VW-treated plants was not significantly different from that of the RI+VW-treated plants. The uptake of C, K, and Ca in the shoots significantly increased in the GM+VW-treated plants when compared to the RI+VW-treated plants (Table 2).

### Mycorrhizal dependency

**Figure 2:**
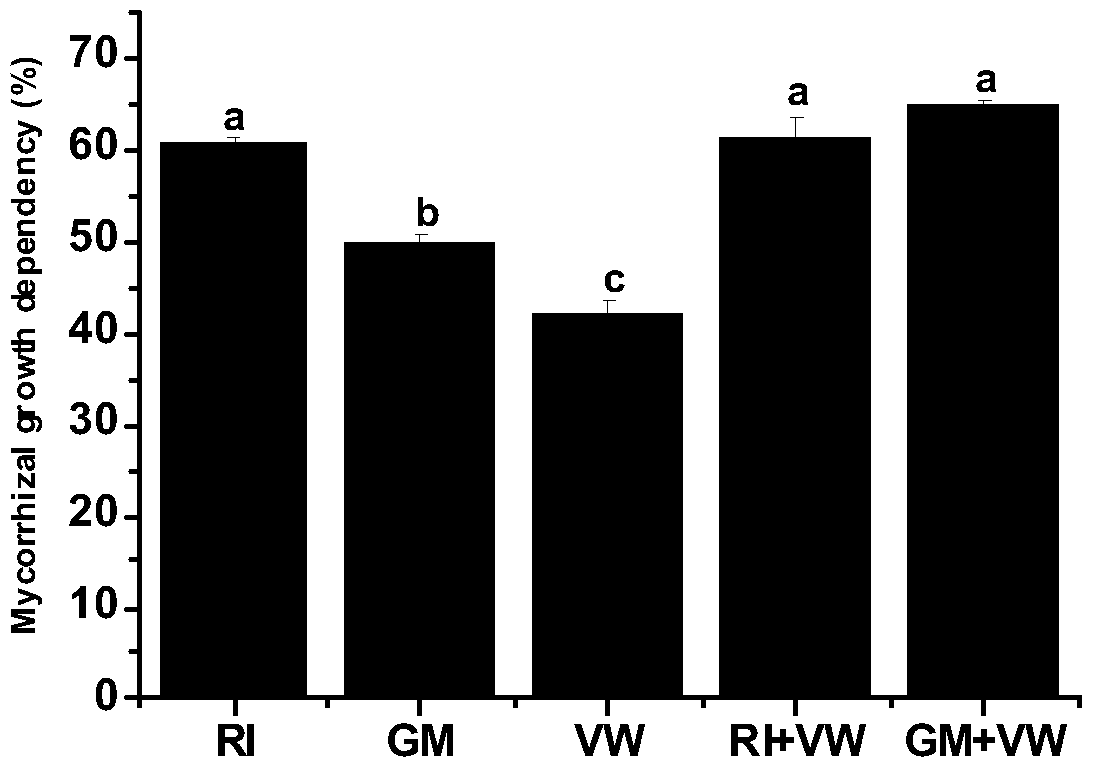
Mycorrhizal growth dependency of *C. assamicum*. RI- *Rhizophagus irregularis,* GM- *G. mosseae,* VW- vermiwash. Values are means of nine biological replicates. Bar shows SEM. Different letters on the column represent significant difference (*p* < 0.05) based on LSD test.

It was observed that the mycorrhizal growth dependency (MGD) of the RI-treated plants was significantly higher than that of the GM-treated plants, followed by the VW-treated plants. VW treatment significantly influenced the MGD in plants. The MGD of the RI treatment was not significantly different from that of the RI+VW treatment. Nevertheless, MGD of the GM+VW treatment was significantly higher than that of the RI treatment, followed by the GM and VW treatments. Analysis of the AMF-VW growth inter-dependency for the dual treatments in comparison to the individual treatments indicated that plants exposed to RI treatment were more dependent on that AMF for growth, while the GM-treated plants were dependent on both the GM and VW for growth (Figure 2).

**Figure 3:**
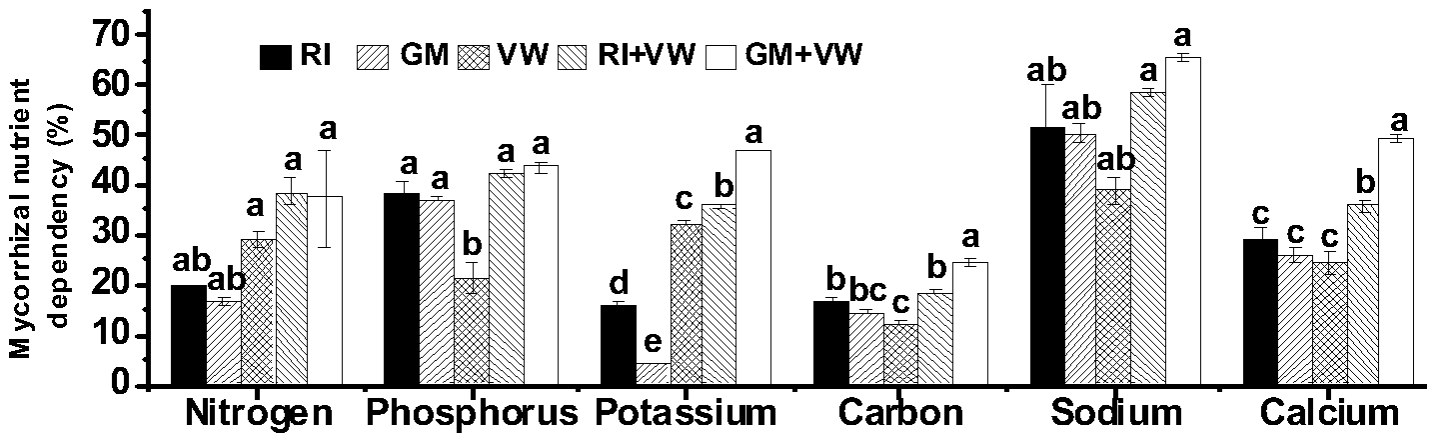
Mycorrhizal shoot nutrient dependency of *C. assamicum*. RI- *Rhizophagus irregularis,* GM- *G. mosseae,* VW- vermiwash. Values are means of nine biological replicates. ± Bar shows SEM. Different letters on the column represent significant difference (*p* < 0.05) based on LSD test.

The inoculation of VW considerably influenced shoot N, K, and C dependencies of the test plants on two AMF, which was evident from the significantly variable response of the plants to the AMF under the VW treatment. Overall, the N and K dependencies of the plants were higher under the VW treatment when compared to those under the AMF treatment; the P, C, and Na dependencies were higher under the AMF treatment when compared to those under the VW treatment (Figure 3).

Furthermore, shoot Ca under the VW treatment appeared to influence the plant’s response to AMF. However, the response of shoot Ca to AMF had lesser prominence than that observed for shoot N, K, and C. Overall, the nutrient dependency data indicate that, although the VW treatment considerably influenced plant-AMF interactions, the RI treatment affected nutrient dependencies to a lesser extent than that by the GM+VW treatment (Figure 3).

### Nutrient stoichiometry

**Figure 4:**
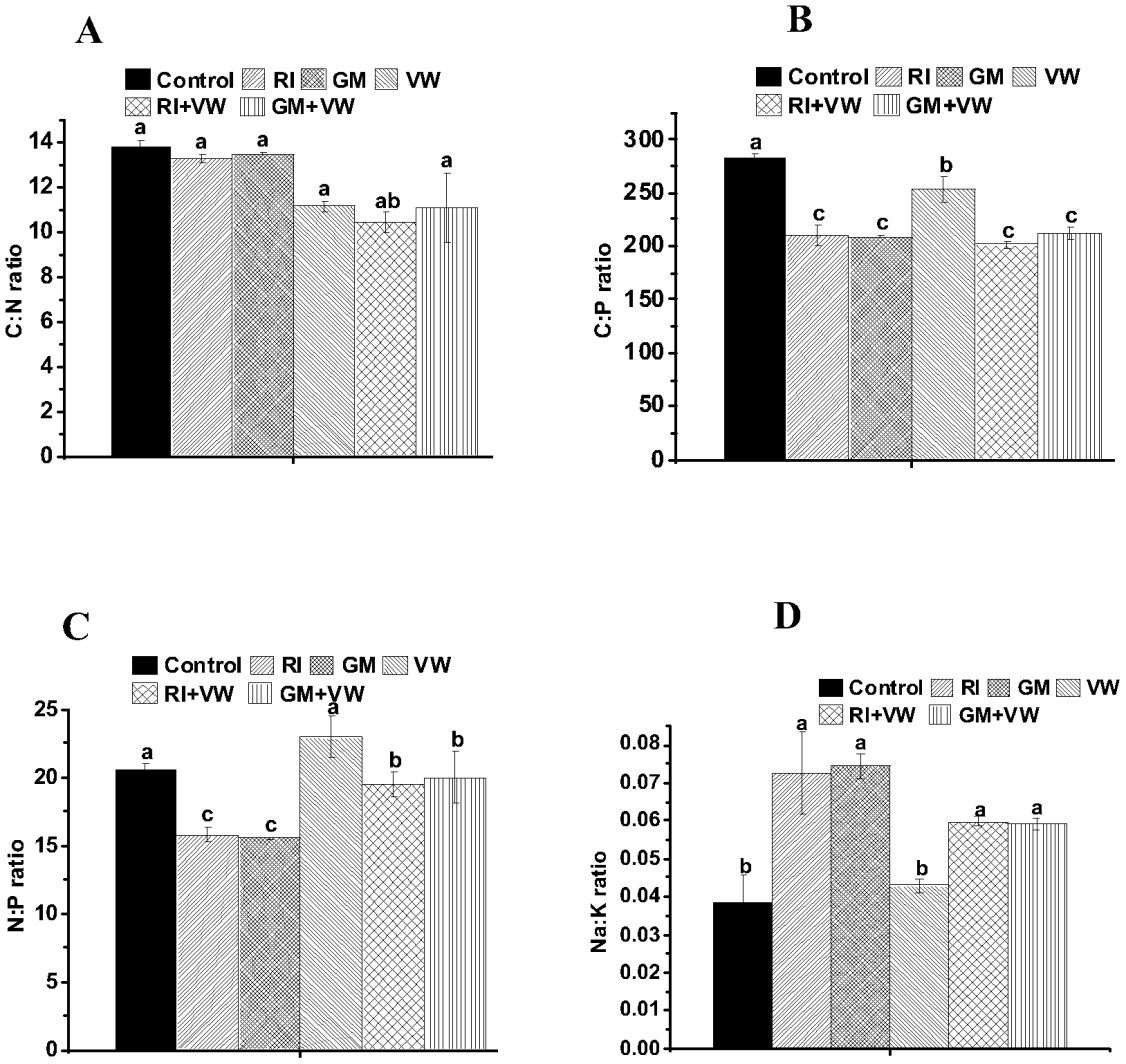
Mycorrhizal nutrient stoichiometries of *C. assamicum* (A-D). RI- *Rhizophagus irregularis,* GM- *G. mosseae,* VW- vermiwash. Values are means of nine biological replicates. Bar shows SEM. Different letters on the column represent significant difference (*p* <0.05) based on LSD test.

With regard to the individual treatments, both the RI- and GM-treated plants showed significantly decreased shoot C:P and N:P ratios and an increased shoot Na:K ratio; however, no significant effect on the C:N ratio was observed when compared to that in the control (Figures 4b&c). The VW-treated plants showed a significant decrease only in the shoot C:N and C:P ratios; there was no significant effect on the Na:K and N:P ratios when compared to those in the control. In the dual treatments, both the GM+VW- and the RI+VW-treated plants showed a significant decrease in the shoot C:P ratio, but there was no significant effect on the stoichiometries of C:N and N:P when compared to those in the control. However, the shoot Na:K ratio increased significantly only in the RI+VW treated plants when compared to that in the control (Figure 4).

A comparison of single RI and GM treatments indicated that there were no significant differences with respect to nutrient stoichiometry. However, a comparison of the RI and VW treatments revealed significantly greater C:P and N:P ratios under the VW treatment and a significantly greater Na:K ratio under the RI treatment. Similarly, the C:P and N:P ratios were significantly greater under the VW treatment when compared to those under the GM treatment; however, the Na:K ratio was significantly higher under the GM treatment.

A comparison between the single and dual treatments exhibited considerable and significant variation in nutrient stoichiometry. The C:N ratio was significantly higher in the individual AMF-treated plants when compared to the RI+VW-treated plants, whereas the N:P ratio was significantly higher in the GM+VW-treated plants when compared to the individual AMF-treated plants. On the other hand, the C:P ratio was significantly higher in the VW-treated plants when compared to the AMF+VW-treated plants. With regard to the two dual treatments, no significant differences were recorded across all nutrient stoichiometric parameters. Overall, the VW application significantly influenced the pattern of nutrient stoichiometry in the plants that were differentially subjected to the two AMF treatments (Figure 4).

## Discussion

### Mycorrhizal colonization, plant growth, and nutrient uptake

The aim of the present study was to evaluate the influence of a VW foliar spray on the mycorrhizal growth dependency and nutrient stoichiometry of *Capsicum assamicum* inoculated using two different AMF in acidic soil under pot conditions. Our results suggest that 100% mycorrhizal colonization was achieved following completion of the experiment, thus, indicating the positive response of *C. assamicum* to the inoculations of the two different AMF. Although several studies have demonstrated differences in the responses of mycorrhizae to crops [7], [8], [37], mycorrhizal colonization and spore count in rhizospheric soil in the current study were not significantly different from the plants inoculated using the two different fungi. Furthermore, the influence of the VW treatment on mycorrhizal colonization could not be inferred as there was no significant difference between the single and dual treatments.

Our results suggest that plant growth and nutrient uptake significantly increased under the AMF and VW treatments, which is in agreement with earlier reports [2], [3], [7]. The positive influence of the VW on plant growth, as observed in the current study, might be ascribed to the presence of plant-growth-promoting substances in the VW, such as humic, fulvic, and other organic acids [4]; auxin-like substances [5]; and cytokinin-like substances [6]. In addition, the absorption of nutrient elements from water solutions applied to the leaves and their beneficial physiological effects on plants have been previously reported [38]. The leaf cuticle is a hydrophobic layer comprised of high molecular-weight biopolymers such as cutin and suberins and hydrophobic C14–C27 epiculticular waxes [39]. Further, recent physiological studies have identified polar aqueous pores, which facilitate the absorption of charged ions into the epidermal cells [40]. The foliar application of fertilizers has become more prevalent agricultural crop production because it is more environmentally friendly when compared to soil fertilization techniques [41].

In addition, in the current study, it was observed that the combined inoculation of AMF and VW had a significantly more positive effect on plant growth and nutrient uptake. Several studies have suggested that combined treatments of AMF and organic amendments may be more beneficial, thus, indicating a positive interaction between these components [42], [43]. In our study, a synergistic interaction between AMF and VW treatments was observed, which might have contributed to enhanced growth and nutrient uptake. To the best of our knowledge, this is the first study on the synergistic interaction of AMF and VW treatments in chili plants.

Interestingly, the VW treatment influenced the extent of the increase in growth and nutrient uptake in plants exposed to the dual treatments. The influence of the VW on the magnitude of this increase was more prominent in the GM+VW treatment when compared to that in the RI+VW treatment. It has been shown that the changes in crop growth and yield in response to soil amendments could be the result of the differential effects of the types of amendment on the proliferation of different AMF [44]. Although we did not conduct a microbiological analysis of the VW used in the current study, it is well known that VW contains macro- and micronutrients such as N, P, K, Ca, Fe, and Mn [3] and several beneficial microbes [45]. These properties of VW may have an indirect role in improving fertility and the physical and chemical conditions of the soilless medium, thus, influencing nutrient availability and contributing to the enhanced growth, yield, and antioxidant content of plants [13], [46]. However, the differential response of plants to the different treatment conditions, as observed in the current study, requires further investigation to ascertain the underlying mechanisms.

### Mycorrhizal dependency

Mycorrhizal dependency, which refers to the degree that a plant benefits from AMF associations [47], has often been quantified by calculating the yield or shoot dry matter ratio between mycorrhizal plants and control plants grown in a particular soil [37], [48]. In the current study, we demonstrated that the application of VW significantly influenced mycorrhizal dependency. It was also observed that the plants inoculated using RI+VW were more dependent on the AMF for growth, while the GM+VW-treated plants were dependent on both the GM and VW for growth. With respect to mycorrhizal nutrient dependency, the VW treatment appeared to influence plant responses to AMF, which was more prominent with regard to shoot N, K, and C concentrations. This may be explained by the fact that the AM symbiotic relationships and subsequent benefits to the host plant are affected by the availability/limitation of nutrients [49]. Moreover, it has been well documented that the symbiotic relationship between AMF and the host plants is not specific; however, under stress, there may be combinations of AM–host plants that are more beneficial for growth [50]. In the current study, nutrient supplementation in the form of foliar spray may have modified the degree of functional plant–AMF associations in acidic soil, which requires further exploration for ascertaining the underlying mechanisms. Interestingly, greater K dependency was observed in VW-treated plants when compared to that in AMF-treated plants, which was shown to increase significantly in the dual treatments. It has been well established that K plays a key role in osmoregulation, stomatal regulation, photosynthesis, and cell extension [37]. In addition, higher K uptake can help mitigate salt stress in plants [51]. Therefore, it can be assumed that the application of VW in combination with AMF may be more beneficial to chili crops grown under conditions of salt stress.

### Nutrient stoichiometry

Ecological stoichiometry is the study of the mass balance of multiple chemical elements in living systems [18], [52], which has implications for nutrient cycling, and the functioning of living organisms and communities of organisms in aquatic and terrestrial ecosystems [17], [53]. The importance of the relative proportions of elements essential for the growth performance of plants has long been known to the farmers. However, there is considerable variability with regard to the optimal ratios for the observed values [20]; thus, its significance has not been widely appreciated among applied researchers. The most investigated relationshiphas been that of C:N:P because N and P commonly limit growth [54]. In addition, while C provides the structural basis for plants, it constitutes a stable percentage (i.e., 50%) of a plant’s dry mass andcan act as a limiting element. These three elements are strongly coupled by their biochemical functions [20]. In the current study, the C:P ratio significantly decreased in all treated plants when compared to that in the control. Similar findings have been reported by Guo et al. [55] in maize plants inoculated using *G. mosseae* and *G. versiforme*. In our study, comparisons among the single treatments revealed significantly greater C:P and N:P ratios under the VW treatment when compared to those under the AMF treatment. This pattern indicates that there was an increase in the uptake of N and a decrease in the uptake of P in the plants treated using VW when compared to those treated using the AMF. The pattern of change in the N:P ratio has been reported as a consequence of AMF-dependent modifications in plant and soil nutrient relations [56]. However, in the current study, the VW treatment appeared to contribute more N to plants when compared to the AMF treatment, thus, leading to changes in the C:N:P stoichiometry of the plant shoots. This pattern was also supported by the fact that the N:P ratio significantly increased using the dual treatments when compared to that in the single treatments of the two AMF. N is an essential component of all enzymes and P is necessary for rRNA synthesis [57]. Therefore, it is likely that the consequential changes in N and P concentrations will affect the allocation of nutrients to a plant’s cellular components, life history strategies, and physiological functions [58]. This could partially explain our findings indicating that the shoot dry matter and shoot P were not significantly affected in the VW- and GM-treated plants, whereas shoot N was significantly higher in the VW-treated plants. Overall, there was considerable variation in the C:N:P stoichiometry of plants receiving the single and dual treatments, thus, indicating the possible effects of VW on plants. However, further detailed studies on nutrient acquisition mechanisms from different sources are required to clearly understand nutrient stoichiometry in plants.

## Conclusions

Overall, the present study clearly indicates that the foliar spray of *C. assamicum* with VW and AMF inoculation in the soil improves plant growth and nutrient uptake under pot conditions. In addition, when combined with AMF treatment, the VW foliar spray significantly and differentially influences the growth and nutrient stoichiometry of plants. The influence of the VW treatment was more pronounced using the GM+VW treatment when compared to that of the RI+VW treatment. Interestingly, the VW treatment appeared to contribute more N to plants than that under the AMF treatment, thus, leading to changes in the C:N:P stoichiometry of the plant shoots. Furthermore, increased K dependency observed under the dual treatments suggests the significance of these treatments in improving crop growth under salt stress. These findings have important implications for selecting effective nutrient sources for crop growth. Future studies, by using advanced techniques such as continuous flow-isotope ratio mass spectrometry, are necessary for investigating the nutrient acquisition mechanisms of plants from different sources and for further precise understanding of the patterns observed in the current study.
